# SARS-CoV-2 seroprevalence among employees of a university hospital in Belgium during the 2020 COVID-19 outbreak (COVEMUZ-study)

**DOI:** 10.1017/S0950268821001540

**Published:** 2021-07-05

**Authors:** Deborah De Geyter, Ellen Vancutsem, Sven Van Laere, Denis Piérard, Patrick Lacor, Ilse Weets, Sabine D. Allard

**Affiliations:** 1 Clinical Biology, Laboratory of Microbiology and Infection Control, Universitair Ziekenhuis Brussel, Brussels, Belgium; 2 Biostatistics and Medical Informatics, Faculty of Medicine and Pharmacy, Vrije Universiteit Brussel, Brussels, Belgium; 3 Internal Medicine and Infectiology, Universitair Ziekenhuis Brussel, Brussels, Belgium; 4 Clinical Biology, Laboratory of Clinical Chemistry, Universitair Ziekenhuis Brussel, Brussels, Belgium

**Keywords:** COVID-19

## Abstract

Between 19 May and 12 June 2020, employees of the UZ Brussel were recruited in this study aiming to document the severe acute respiratory syndrome coronavirus 2 (SARS-CoV-2) seroprevalence, to investigate the potential work-related risk factors for SARS-CoV-2 infection and to estimate the proportion of asymptomatic infections. In total, 2662 participants were included of whom 7.4% had immunoglobulin G antibodies against SARS-CoV-2. Of the participants reporting a positive polymerase chain reaction for SARS-CoV-2, 89% had antibodies at the time of blood sampling. Eleven per cent of the antibody positive participants reported no recent symptoms suggestive of coronavirus disease 2019 (COVID-19). Participants reporting fever, chest pain and/or anosmia/ageusia were significantly more frequently associated with the presence of antibodies against SARS-CoV-2. The presence of antibodies was highest in the group that had had contact with COVID-19-infected individuals outside the hospital with or without using appropriate personnel protective equipment (PPE) (*P* < 0.001). Inside the hospital, a statistically significant difference was observed for the employees considered as low-risk exposure compared to the intermediate-risk exposure group (*P* = 0.005) as well as the high-risk exposure group compared to the intermediate exposure risk group (*P* < 0.001). These findings highlight the importance of using correct PPE.

## Introduction

A novel zoonotic coronavirus was discovered in Wuhan (Hubei Province, China) in mid-December 2019 and was named severe acute respiratory syndrome coronavirus 2 (SARS-CoV-2). The virus rapidly spread to the rest of the world, including Europe. It particularly affects the respiratory system, generating coronavirus disease 2019 (COVID-19) [1]. The spectrum of symptomatic COVID-19 ranges from mild to critical. Mild cases present fever and mild respiratory symptoms. Severe illness encompasses respiratory distress with decreased oxygen saturation, while critical cases can present respiratory failure with need for mechanical ventilation, shock and/or multi-organ failure. Asymptomatic infections have also been observed, but their proportion remains unclear [2–4].

The routes of transmission of SARS-CoV-2 are not yet completely clarified. Although human-to-human transmission of the virus occurs mainly through close contact and droplets, airborne precautions are recommended in certain situations. Despite appropriate personal protective equipment (PPE), health care workers (HCWs) are at high risk of infection as they work in close contact with COVID-19 patients. Appropriate PPE includes gown, gloves, eye protection and a surgical mask. At the time of the study, the use of filtering face piece particles (FFP)-2, together with a face shield were recommended when performing aerosol-generating procedures, such as high-flow oxygen administration, non-invasive ventilation and tracheal intubation. Special attention is needed concerning the sequence of putting on and taking off the PPE at all times, despite periods of high work pressure [5, 6].

UZ Brussel employees presenting symptoms suggestive of COVID-19 are offered to be tested for the presence of viral RNA with real-time polymerase chain reaction (PCR) on nasopharyngeal swabs, which is considered as the gold standard for diagnosis of the virus. The sensitivity of that technique is however not 100% due to sampling errors and undetectable viral loads in mild cases or when sampling occurs too early or too late in the disease progression [7]. The SARS-CoV 2003 epidemic demonstrated that serological assays were a useful diagnostic tool of non-acute infections [8]. For that reason, detection of antibodies against SARS-CoV-2 has been proposed as a diagnostic test for patients suspected of COVID-19 in whom no respiratory sample was taken at the time of acute illness or in patients in whom an asymptomatic, atypical or mild infection appeared [9].

Immunoglobulins (Ig) M (IgM) and G (IgG) against SARS-CoV-2 are detectable within 1–14 days after onset of symptoms with a low cost and easy to perform blood tests [10–12]. A Chinese study investigated the serological response in 285 patients with COVID-19. They showed that within 19 days of symptoms, 100% of patients tested positive for IgG and seroconversion for IgG and IgM occurred simultaneously or sequentially [13]. Another paper demonstrated that the median duration of IgG detection was 14 days after symptom onset, with a positive rate of 77.9% in confirmed and probable cases of COVID-19 [12].

Although it is still uncertain whether convalescing patients have a risk of re-infection and the correlation between protection against SARS-CoV-2 and the antibody longevity remains unclear, recent data suggest that SARS-CoV-2 antibodies could protect at least for some time from subsequent viral exposures [14]. Studies in severely ill individuals have identified robust cellular and humoral immune responses against the virus. Asymptomatic infection with SARS-CoV-2 has also been described, but it is unknown whether this is sufficient to develop antibody responses [15].

Recently, a chemiluminescent microparticle immunoassay for the qualitative detection of IgG in human serum or plasma against the SARS-CoV-2 nucleocapsid on the Architect platform (Abbott) has shown excellent analytical performance [16].

Consequently, determination of the SARS-CoV-2 serological status among HCWs has several advantages: (1) the epidemiology of SARS-CoV-2 infection among HCWs at high risk of infection can be evaluated and compared with that of non-HCW employees at lower risk of infection, (2) a major concern of HCWs is the risk of contracting the disease. The PPE measures applied are therefore sometimes disproportionate to the risk encountered. Determination of the SARS-CoV-2 serological status among employees will be helpful to review the preventive measures, including PPE, that are being applied in the different units of the hospital, (3) knowledge on SARS-CoV-2 serostatus might positively influence the well-being of the HCWs (a negative serostatus confirms the efficacy of the PPE measures, a positive serostatus most probably confers protective immunity).

We aimed to prospectively document the SARS-CoV-2 seroprevalence among employees of the UZ Brussel, both medical and paramedical staff as well as administrative and technical staff. In addition, we wanted to investigate potential work-related risk factors for SARS-CoV-2 infection and to estimate the proportion of asymptomatic infections among employees of the UZ Brussel.

## Methods

### Study design

Ethical approval for this study was obtained from the ethical committee of UZ Brussels (BUN 2021430000091).

Participants were included between 19th May and 12th June 2020. All employees, including those with previously confirmed COVID-19, currently working at the UZ Brussel, were eligible for inclusion in this monocentric interventional prospective cohort study, named the COVEMUZ-study.

Since the COVID-19 outbreak, all employees of the UZ Brussel experiencing respiratory symptoms suggestive of COVID-19 are tested using real-time PCR. If positive, they are isolated at home for a minimum of 7 days, if negative and if the clinical state allows it, they can come to work wearing a mask. Those who recovered from a documented infection are also allowed to work. If a participant develops symptoms, he/she will follow the recommended standard of care and will be tested by using PCR on nasopharyngeal swabs in parallel.

After signing written informed consent, participants were recruited for blood sampling and completion of a pseudonymised questionnaire using Qualtrics Survey Software on demographic characteristics, job function, risks of exposure to SARS-CoV-2 and symptoms experienced between 1 March 2020 and the time of study inclusion. Symptoms are chosen as described by the Belgian Public Health department (Sciensano) in their guidelines for case definition and testing (Sciensano, Public Health Institute Belgium, Case_Definition_Testing_NL.pdf, https://covid-19.sciensano.be/nl/covid-19-gevalsdefinitie-en-testing; Version 23, November 2020).

Sample storage and analysis were performed at the laboratory of Microbiology of the UZ Brussel. Anti-SARS-CoV-2 IgG antibodies were detected in sera using the Alinity™ i system (Abbott, Illinois, USA) according to the manufacturer's instructions; where 1.40 was recommended as a cut-off to define the presence of SARS-CoV IgG antibodies against the nucleocapsid.

### Inclusion and exclusion criteria

Any adult employee of the UZ Brussel who provided signed informed consent to participate in the study was eligible for inclusion.

Staff not active during the inclusion period were excluded.

### Statistical analysis

Descriptive statistics were provided in terms of absolute and relative frequencies. The seroprevalence 95% confidence interval (CI) was calculated by the asymptotic method. Odds ratios (ORs) and 95% CIs were calculated using bivariable logistic regression for explaining relationships with exposure risks, i.e. their risk group and contact with COVID-19-positive individuals both in- and outside the hospital. Positive seroprevalence was modelled by including the covariates risk group, contact inside the hospital and contact outside the hospital one by one. Contrasts of ordinal variables were specified such that ascending categories were compared. Additionally, to compare exposure risk from having patient contact inside the hospital to having contact with COVID-19-positive individuals outside the hospital, the risk of seroprevalence was also determined in function of the interaction of having contact in- and/or outside the hospital. Furthermore, multivariable logistic regression was used to assess the relationship between symptoms questioned in the survey of the employee and having a positive seroprevalence. All these effects were graphically plotted in terms of ORs and their 95% CI by means of forest plots. A result was found to be statistically significant when having *P* values lower than 0.05. To correct the significance level for multiple testing, a Benjamini–Hochberg correction was applied. Analyses were performed using RStudio running on R version 4.0.2.

## Results

In total, 2662 participants were included in this study: 75.4% women, 24.6% men and <0.01% other (Table 1). Medical doctors counted for 16.7%, 33.4% were nurses, 49.9% had another (para)medical profession and 23.1% had a non-medical occupation, such as technical, catering and administrative staff. The majority of the participants (48%) were in the age category of 30–50 years, followed by older than 50 years (31.5%) and younger than 30 years (21.5%).

**Table 1.** Characteristics of the employees of the UZ Brussels included in the study

	N	%
Gender		
Men	653	24.5
Women	2008	75.4
Other	1	<0.01
Age		
<30	545	20.5
30–50	1278	48.0
>50	839	31.5
Job function		
Medical doctor	445	16.7
Nurse	863	32.4
(Para)medical	738	27.7
Other	616	23.1
Risk group		
Low risk	1141	42.9
Intermediate risk	877	32.9
High risk	644	24.2
Exposure to confirmed cases in the hospital		
No	1236	46.4
Yes with PPE	996	37.4
Yes without PPE	430	16.2
Exposure to confirmed cases in the hospital		
No	2418	90.8
Yes with PPE	90	3.4
Yes without PPE	154	5.8
	N = 2662	

**Fig. 1.**
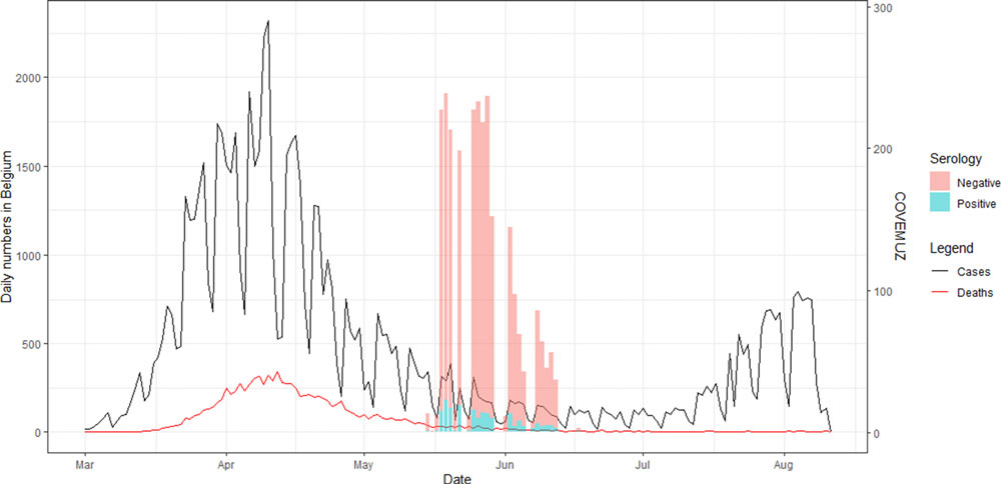
Overview of timing and seroprevalence of the study in comparison with COVID-19 epidemiology (number of cases and deaths) in Belgium. The study was performed between May 19th and June 12th 2020. Seropositive employees are shown in blue, seronegative ones in pink.

Overall, IgG antibodies against SARS-CoV-2 were identified in 198/2662 employees (7.4%; 95% CI: 6.4–8.4). Interestingly, of the patients recalling a recent positive PCR (*N* = 91) for SARS-CoV-2 on nasopharyngeal swabs, 11% had no antibodies at the time of blood sampling. In Figure 1, an overview of timing and seroprevalence of the study in comparison with COVID-19 epidemiology (number of cases and deaths) in Belgium is shown.

**Fig. 2.**
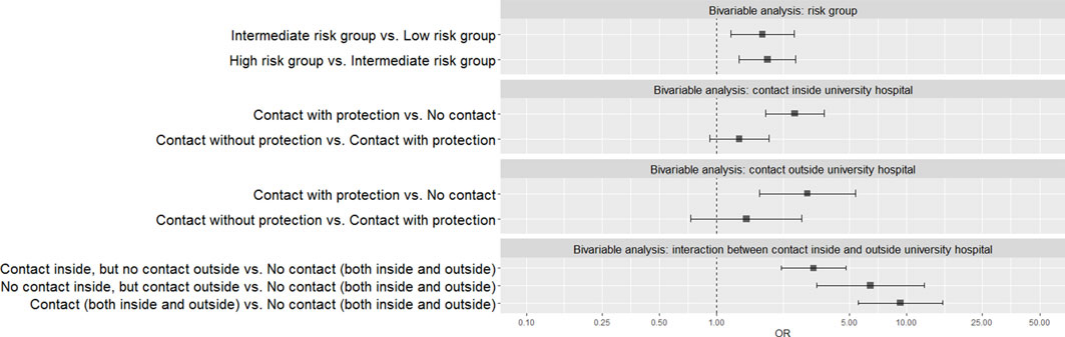
Forest plot of bivariable models demonstrating the ORs with its 95% CI comparing the exposure variables in ascending order of risk to get infected with COVID-19.

Risk contact inside and/or outside the hospital, as retrospectively documented in the questionnaire, allowed us to categorise participants in three groups: (1) presumed to have had no risk contact with COVID-19-suspected or confirmed individuals (no contact); (2) presumed to have had contact with COVID-19-suspected or confirmed individuals, but with the recommended PPE (contact with protection) and (3) presumed to have had contact with COVID-19-suspected or confirmed individuals, without PPE (contact without protection). The majority of respondents was categorised as belonging to the low-risk group (42.9%) and indicating they presumed to have had no contact with COVID-19-suspected or confirmed individuals both inside (46.4%) and outside the hospital (90.8%) (Table 1). Contact without protection, whether the contact was inside or outside the hospital, was associated with a statistically significant higher likelihood of having been infected with SARS-CoV-2 (OR = 2.579 and 3.004, respectively, *P* < 0.001) (Tables 2 and 3; Fig. 2). Although the likelihood of having been infected with SARS-CoV-2 was higher for participants recalling contacts without protection compared to contact with protection, the differences were not statistically significant (Table 3).

**Table 2.** Presence or absence of SARS-CoV-2 IgG antibodies according to risk group and exposure to confirmed COVID-19 patients

	Absence of SARS-CoV-2 IgG antibodies N (%)	Presence of SARS-CoV-2 IgG antibodies N (%)
Risk group		
Low risk	1091 (95.6)	50 (4.4)
Intermediate risk	812 (92.6)	65 (7.4)
High risk	561 (87.1)	83 (12.9)
Exposure to confirmed cases in the hospital		
No	1187 (96.0)	49 (4.0)
Yes with PPE	900 (90.4)	96 (9.6)
Yes without PPE	377 (87.7)	53 (12.3)
Exposure to confirmed cases outside the hospital		
No	2269 (93.8)	149 (6.2)
Yes with PPE	75 (83.3)	15 (16.7)
Yes without PPE	120 (77.9)	34 (22.1)

**Table 3.** Logistic regression with seroprevalence as dependent variable and exposure risks (composed of their job function and answer of the respondent) as independent variable

		95% CI		
Symptoms in relation to seroprevalence	OR	Lower	Upper	Adjusted *P* value
Risk group				
Intermediate-risk group *vs.* low-risk group	1.745	1.193	2.550	0.004
High-risk group *vs.* intermediate-risk group	1.854	1.317	2.610	<0.001
Contact inside university hospital				
Contact with protection *vs.* no contact	2.579	1.809	3.677	<0.001
Contact without protection *vs.* contact with protection	1.319	0.924	1.884	0.127
Contact outside university hospital				
Contact with protection *vs.* no contact	3.004	1.685	5.355	<0.001
Contact without protection *vs.* contact with protection	1.436	0.733	2.811	0.292
Interaction between contact inside and outside university hospital				
Contact inside, but no contact outside *vs.* no contact (both inside and outside)	3.242	2.192	4.795	<0.001
No contact inside, but contact outside *vs.* no contact (both inside and outside)	6.443	3.362	12.348	<0.001
Contact (both inside and outside) *vs.* no contact (both inside and outside)	9.250	5.547	15.424	<0.001

For being able to observe the direct impact of the location where a hospital staff member had contact with COVID-19-positive individuals (inside or outside the hospital), an interaction between both risk factors (inside contact and outside contact) was used. Since no statistically significant difference was observed between contact with and contact without protection in previous analyses, the interaction was only made for having no contact or having contact for both inside and outside the hospital (i.e. regrouping groups 2 and 3 from above as one category) (presumed to have had contact with COVID-19-suspected or confirmed individuals). When observing staff members that did not have contact with suspected or confirmed individuals to persons that did have contact inside the hospital without presuming to have contact outside, we observe that having contact increased the likelihood of having antibodies (OR = 3.242, Table 3). When presuming to have only contact outside the hospital, this risk on average nearly doubled (OR = 6.443) compared to the reference group. When presuming to have contact both inside and outside the hospital, this risk now marginally increased (OR = 9.250). All differences compared to hospital staff having no contact were found statistically significant (*P* < 0.001). The additional risk between having contact outside and having contact inside the hospital was statistically significant (*P* = 0.029).

Next, based on the work-related SARS-CoV-2 exposure, participants were categorised into three risk groups, ranging from low to high (see Table 4).

**Table 4.** Definition of risk groups according to patient contact

Risk group	Description
Low risk	Daily non-physical contact with patients without known or suspected COVID-19 on non-COVID-19-designated wards or without patient contact
Intermediate risk	Daily physical contact with patients without known or suspected COVID-19 on non-COVID-19-designated wards or with daily non-physical contact with known or suspected COVID-19 patients (e.g. hospital porter and cleaning operative) on COVID-19-designated wards
High risk	Daily physical contact with known or suspected COVID-19 patients on COVID-19-designated wards

In the bivariable analyses observing differences in variables related to exposure, the variables risk group, contact inside hospital and contact outside hospital were used as independent variables (see Table 2 and Fig. 2). The likelihood of having COVID-19 IgG antibodies was statistically higher for both the intermediate- and high-risk group compared to the low-risk group (OR = 1.745, *P* = 0.004; OR = 1.854, *P* < 0.001, respectively) (Table 3).

**Fig. 3.**
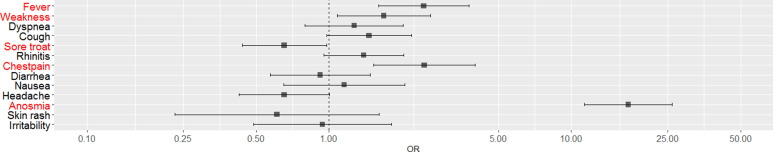
Forest plot of multivariate model with symptoms demonstrating the ORs with its 95% CI.

Eleven per cent (22/198, 11.1%) of the participants with positive antibodies did not recall symptoms suggestive of COVID-19. Recent fever, weakness, chest pain and/or anosmia/ageusia were associated with a significantly higher likelihood of having SARS-CoV-2 IgG antibodies (OR = 2.457, 1.682, 2.474 and 17.182, respectively; Table 5 and Fig. 3). In contrast, a sore throat was associated with a lower likelihood of having SARS-CoV-2 IgG antibodies (OR = 0.653; *P* = 0.096). After correction for multiple testing, the symptoms fever, chest pain and anosmia/ageusia were statistically significantly associated with the presence of SARS-CoV-2 IgG antibodies (Table 5). Sixty-five per cent of the participants who had a combination of fever and/or anosmia/ageusia had antibodies against SARS-CoV-2. Participants recalling fever and or anosmia/ageusia were 50% more probable to develop antibodies.

**Table 5.** Logistic regression with seroprevalence as dependent variable and symptoms questioned as independent variable

		95% CI		
Symptoms in relation to seroprevalence	OR	Lower	Upper	Adjusted *P* value
Fever	2.457	1.600	3.774	<0.001
Weakness	1.682	1.078	2.624	0.072
Dyspnoea	1.268	0.796	2.022	0.414
Cough	1.460	0.975	2.187	0.123
Sore throat	0.653	0.437	0.975	0.096
Rhinitis	1.388	0.948	2.030	0.148
Chest pain	2.474	1.525	4.013	0.001
Diarrhoea	0.919	0.570	1.481	0.788
Nausea	1.154	0.650	2.048	0.739
Headache	0.652	0.425	1.002	0.111
Anosmia/ageusia	17.182	11.342	26.030	<0.001
Skin rash	0.610	0.231	1.612	0.413
Irritability	0.938	0.488	1.804	0.849

## Discussion

We found that 7.4% of the employees of the UZ Brussel were seropositive for SARS-CoV-2 antibodies between 19 May and 12 June 2020. These results are in line with those of the Institute of Public Health in Belgium [17] where 7.7% (22–26 April 2020), 7.8% (6–10 May 2020) and 8.8% (19–24 May 2020) of HCWs in Belgian hospitals developed antibodies. They found that unprotected contact with a confirmed case was the only factor associated with seropositivity. In their study, only employees in direct contact with patients were included, which is in contrast with our study where all employees have been recruited.

In the general population in Belgium, seropositivity ranged from 4.7% (11–13 May 2020), 5.1% (25–27 May 2020) to 4.3% on 8–10 June 2020 [18].

Interestingly, among employees recalling a positive PCR on nasopharyngeal swabs, 11% were antibody negative. This can possibly be explained by the fact that antibodies still had to be developed (for those with a recent positive PCR/COVID-19) [11, 12] or that antibody titres decreased below the threshold for positivity (for those with a positive PCR/COVID-19 in the early beginning of the pandemic) [19].

Another hypothesis is that in people with mild symptoms, the humoral immune response is not sufficient to produce antibodies [14] or that other immune responses, such as the cellular immune system, play a role [20].

Eleven per cent of the employees with positive antibodies had no symptoms at all, confirming that asymptomatic infections indeed occur [2–4]. A pitfall in our study is that recall bias may be present since it concerns a cross-sectional investigation. Fever, weakness, chest pain and anosmia/ageusia were associated with a statistically significant higher likelihood of being.

Our data revealed that the highest risk of being seropositive for COVID-19 was observed in participants that had had contacts with COVID-19-infected people outside the hospital, compared to the ones only having contact with patients inside the hospital.

To evaluate the exposure to SARS-CoV-2 among employees, three risk categories were defined. We found that the risk of having COVID-19-related antibodies was, respectively, 1.745 and 1.854 times higher in the intermediate-risk *vs.* low-risk groups and high-risk *vs.* intermediate-risk groups. These differences were statistically significant. A comparable study performed between 22 April and 30 April 2020 in employees from another Belgian hospital, showed that 6.4% of employees had developed antibodies against SARS-CoV-2 [21]. Being involved in clinical care, having worked during the lockdown phase, being involved in care for patients with COVID-19 and exposure to COVID-19-positive coworkers, were not statistically significantly associated with seroprevalence. In addition and in line with our results, they showed that having a household contact with suspected or confirmed COVID-19 was associated with a significant higher risk (*P* < 0.001) to be antibody positive. Our study population had a slightly more elevated percentage of seropositive status (7.4% *vs*. 6.4%). As we tested somewhat later in the time frame of the pandemic, this difference could be explained by the fact that more employees could have developed antibodies against SARS-CoV-2. They also found that prior anosmia was associated with the presence of antibodies, with an OR of 7.78 (95% CI: 5.22–11.53), as well as fever and cough.

In the beginning of the pandemic, until July, when resources were scarce, HCWs on the corona units in contact with patients were only wearing FFP-2 masks on the intensive care wards, in the operating theatre, or when aerosol-generating procedures such as high-flow oxygen administration, non-invasive ventilation or tracheal intubation were performed. This was in line with the National and the guidelines of the World Health Organization [World Health Organization (2020). Rational use of personal protective equipment for coronavirus disease (COVID-19): interim guidance, 27 February 2020; https://apps.who.int/iris/handle/10665/331215]. However, in June 2020, National guidelines were adapted (Sciensano, Public Health Institute Belgium, https://covid-19.sciensano.be/sites/default/files/Covid19/COVID-19_procedure_hospitals_NL.pdf, Versie 23; November 2020), stating that every HCW in contact with patients in a corona unit should wear an FFP-2 mask. Moreover, when supply is sufficient, every HCW in contact with patients should wear an FFP-2 mask when there is close contact (less than 1.5 m) for more than 15 min or when the patient cannot wear a surgical FFP-1 mask. These new guidelines, together with the fact that we have concluded that the high-risk group of employees developed significantly more antibodies compared to the intermediate- and low-risk group encouraged us to implement these updated guidelines from July in our hospital. However, not only patient contact involves a risk but of major importance is to keep distance between employees and to maximise hygiene precautions during for example breaks to prevent transmission.

Our results are in contrast with those of a German study where they saw that seroprevalence was higher in the intermediate-risk group *vs.* the high-risk group, although this finding was not statistically significant [22]. The difference might be explained by the difference in use of FFP masks or the difference in risk categorisation.

Our study shows that between 19th May and 12th June 2020, the seroprevalence of SARS-CoV-2 was relatively low and in line with the results of other hospitals in Belgium. Risks for being seropositive are close patient contact and wearing less PPE (both inside and outside the hospital). Inside the hospital, HCWs with direct patient contact in a corona ward had the highest risk. This emphasises the important role of adapted PPE in order to prevent the transmission of the virus.

A limitation of the study can be the fact that we used a nucleocapsid-based immuno-assay. It is described that the nucleocapsid antigen decreases over time and only shows a sensitivity of 70% at more than 81 days after infection, suggesting that assays based on that principle are less suitable for longitudinal epidemiological studies compared to assays that detect the spike-protein antibodies [23]. Another study in Belgium [24] showed that the mean half-life of nucleocapsid antibodies was 74.8 days, so even if our study was conducted shortly after the first wave, it can be possible that in healthy and immunocompetent HCWs, the antibodies were no longer detectable.

Another limitation of the study is that the PCR results were recalled and we cannot exclude recalling bias.

## Data Availability

The data that support the findings of this study are available upon request by contacting the corresponding author by email (Deborah.degeyter@uzbrussel.be).
